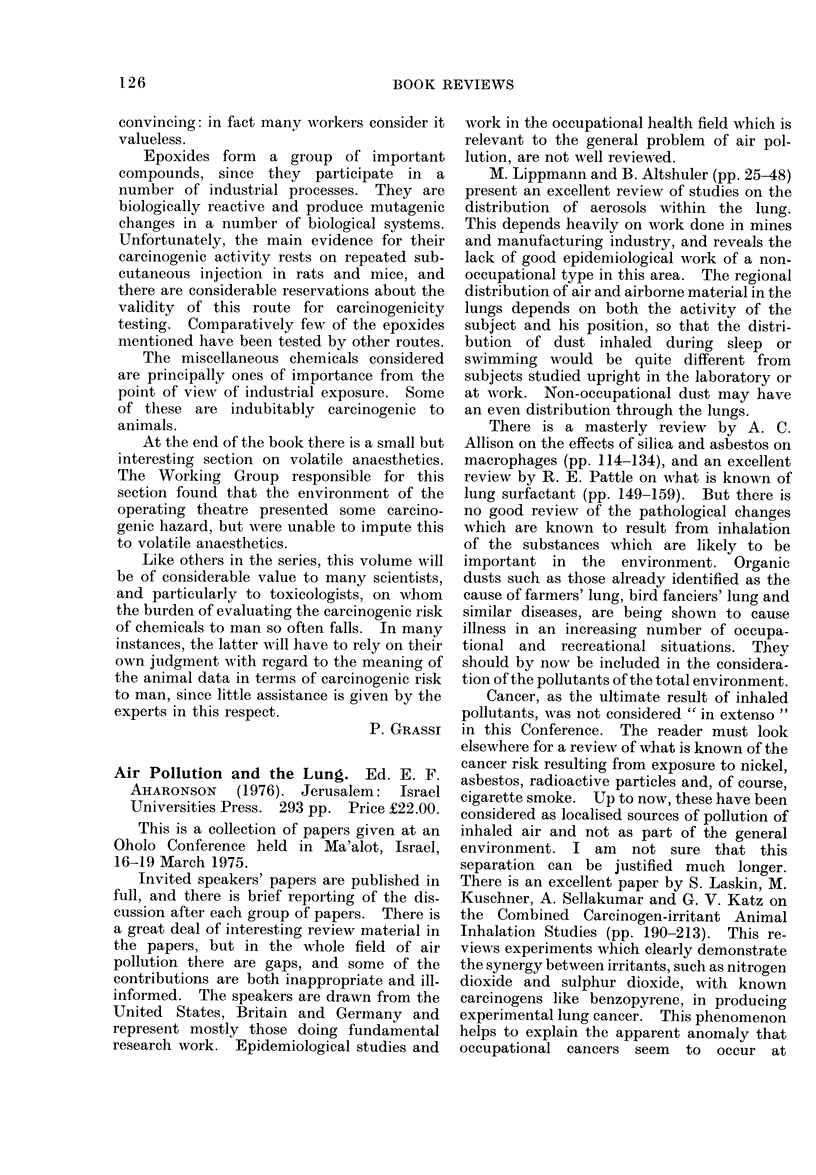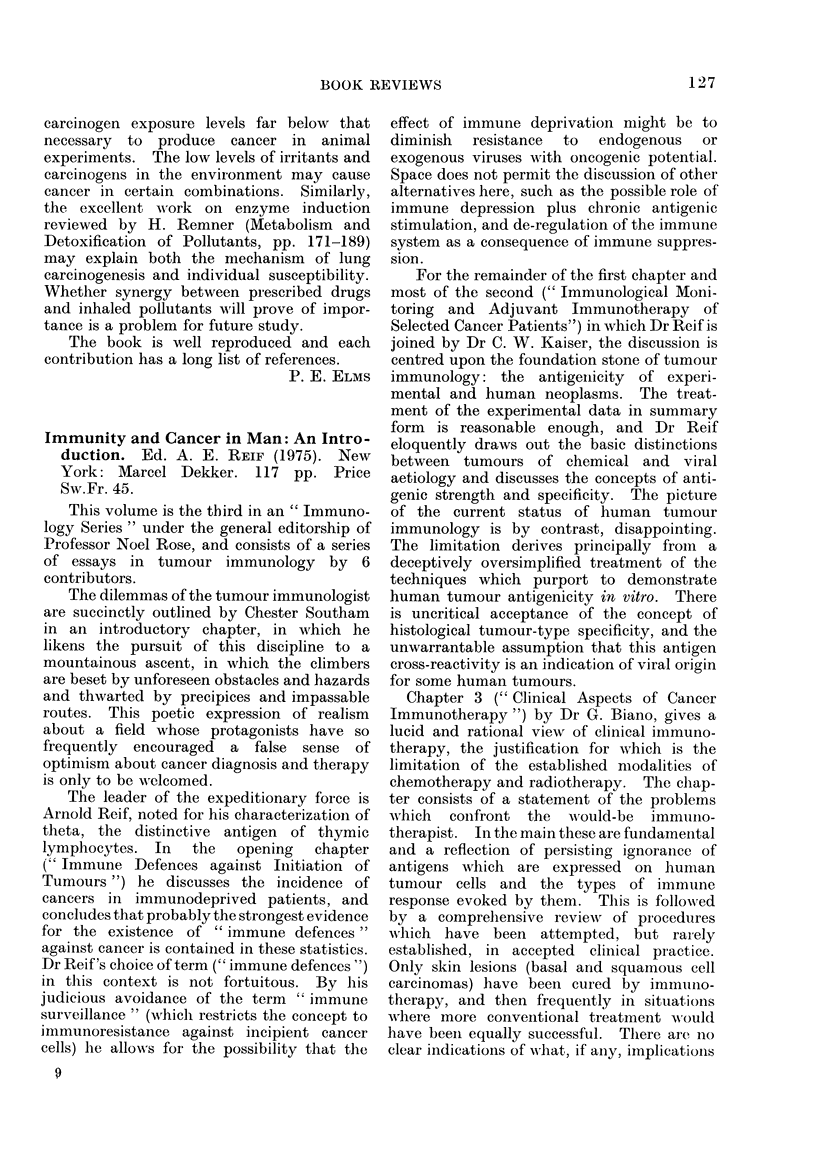# Air Pollution and the Lung

**Published:** 1977-01

**Authors:** P. E. Elms


**Air Pollution and the Lung.** Ed. E. F. Aharonson (1976). Jerusalem: Israel Universities Press. 293 pp. Price £22.00.

This is a collection of papers given at an Oholo Conference held in Ma'alot, Israel, 16–19 March 1975.

Invited speakers' papers are published in full, and there is brief reporting of the discussion after each group of papers. There is a great deal of interesting review material in the papers, but in the whole field of air pollution there are gaps, and some of the contributions are both inappropriate and ill-informed. The speakers are drawn from the United States, Britain and Germany and represent mostly those doing fundamental research work. Epidemiological studies and work in the occupational health field which is relevant to the general problem of air pollution, are not well reviewed.

M. Lippmann and B. Altshuler (pp. 25–48) present an excellent review of studies on the distribution of aerosols within the lung. This depends heavily on work done in mines and manufacturing industry, and reveals the lack of good epidemiological work of a non-occupational type in this area. The regional distribution of air and airborne material in the lungs depends on both the activity of the subject and his position, so that the distribution of dust inhaled during sleep or swimming would be quite different from subjects studied upright in the laboratory or at work. Non-occupational dust may have an even distribution through the lungs.

There is a masterly review by A. C. Allison on the effects of silica and asbestos on macrophages (pp. 114–134), and an excellent review by R. E. Pattle on what is known of lung surfactant (pp. 149–159). But there is no good review of the pathological changes which are known to result from inhalation of the substances which are likely to be important in the environment. Organic dusts such as those already identified as the cause of farmers' lung, bird fanciers' lung and similar diseases, are being shown to cause illness in an increasing number of occupational and recreational situations. They should by now be included in the consideration of the pollutants of the total environment.

Cancer, as the ultimate result of inhaled pollutants, was not considered " in extenso " in this Conference. The reader must look elsewhere for a review of what is known of the cancer risk resulting from exposure to nickel, asbestos, radioactive particles and, of course, cigarette smoke. Up to now, these have been considered as localised sources of pollution of inhaled air and not as part of the general environment. I am not sure that this separation can be justified much longer. There is an excellent paper by S. Laskin, M. Kuschner, A. Sellakumar and G. V. Katz on the Combined Carcinogen-irritant Animal Inhalation Studies (pp. 190–213). This reviews experiments which clearly demonstrate the synergy between irritants, such as nitrogen dioxide and sulphur dioxide, with known carcinogens like benzopyrene, in producing experimental lung cancer. This phenomenon helps to explain the apparent anomaly that occupational cancers seem to occur at carcinogen exposure levels far below that necessary to produce cancer in animal experiments. The low levels of irritants and carcinogens in the environment may cause cancer in certain combinations. Similarly, the excellent work on enzyme induction reviewed by H. Remner (Metabolism and Detoxification of Pollutants, pp. 171–189) may explain both the mechanism of lung carcinogenesis and individual susceptibility. Whether synergy between prescribed drugs and inhaled pollutants will prove of importance is a problem for future study.

The book is well reproduced and each contribution has a long list of references.

P. E. ELMS